# An Evaluation of Dried Blood Spots and Oral Swabs as Alternative Specimens for the Diagnosis of Dengue and Screening for Past Dengue Virus Exposure

**DOI:** 10.4269/ajtmh.2012.11-0713

**Published:** 2012-07-01

**Authors:** Katherine L. Anders, Nguyen Minh Nguyet, Nguyen Than Ha Quyen, Tran Van Ngoc, Ta Van Tram, Tran Thi Gan, Nguyen Thanh Tung, Nguyen Thi Dung, Nguyen Van Vinh Chau, Bridget Wills, Cameron P. Simmons

**Affiliations:** Oxford University Clinical Research Unit, Wellcome Trust Major Overseas Program, Ho Chi Minh City, Vietnam; Centre for Tropical Medicine, University of Oxford, United Kingdom; Department of Epidemiology and Preventive Medicine, Monash University, Melbourne, Australia; Hospital for Tropical Diseases, Ho Chi Minh City, Vietnam; Tien Giang Provincial Hospital, Tien Giang, Vietnam

## Abstract

Non-invasive specimens for dengue diagnosis may be preferable where venous blood is difficult to collect and/or process, such as community-based or remote settings or when sampling from young children. We evaluated the performance of oral swabs and dried blood spots (DBS), compared with plasma, in diagnosing acute dengue and screening for past dengue virus (DENV) exposure. DENV-specific immunoglobulin (Ig) M, IgG, and NS1 antigen were detected both in oral swabs and DBS from acute patients. Oral swabs were less sensitive (IgM: 68.7%, IgG: 91.9%, NS1: 64.7%), but retained good specificity (100%, 92.3%, 95.8%, respectively) compared with plasma. DBS displayed high sensitivity (IgM: 100%, IgG: 96%, NS1: 100%) and specificity (IgM: 75%, IgG: 93%). DENV RNA was amplified from DBS (sensitivity 95.6%) but not from oral swabs. DENV-IgG (indicative of past flavivirus exposure) were detected with moderate sensitivity (61.1%) but poor specificity (50%) in oral swabs from healthy volunteers. Dried blood spots allow sensitive and specific diagnosis of acute dengue by serological, molecular, and antigen detection methods. Oral swabs may be an adequate alternative where blood cannot be collected.

## Introduction

In most dengue-endemic countries, clinical management and epidemiological surveillance of dengue cases is based on a clinical diagnosis, with only a small proportion of cases receiving laboratory confirmation. Greater availability of timely, sensitive, and specific diagnostics for dengue may improve patient management, including the identification and appropriate treatment of patients with diseases other than dengue, and improve the accuracy of surveillance data. Serological assays can detect dengue virus (DENV)-specific immunoglobulin (Ig) M or IgG from around 4–5 days after fever onset. Diagnosis by reverse transcription-polymerase chain reaction (RT-PCR) or, less commonly, virus isolation is possible early in infection but is expensive and is rarely used in routine management. More recently, enzyme-linked immunosorbent assay (ELISA)-based and lateral flow rapid tests for detection of DENV NS1 antigen have been shown to have good specificity for diagnosis of early acute dengue, although their sensitivity was dependent on the infecting serotype and the host humoral immune response.^1^ The sensitivity of all dengue diagnostic assays depends on the timing of sample collection. Venous blood collection is typically required for all of the previous dengue diagnostic methods. Although this is relatively straightforward in a clinical setting, it requires expertise and equipment for sample collection, processing, and storage, which may be lacking in community-level or remote settings. Furthermore, alternatives to venous blood collection may be preferable in certain populations such as young children, or in serosurveillance studies in healthy volunteers.

Oral swabs are painless, non-invasive, and simple to collect, and have been used to detect pathogen-specific antibody in a number of viral infections, including measles, rubella, parvovirus B19, and hepatitis B.^2^–^5^ Viral antigen (hepatitis B surface antigen)^6^ and RNA (measles, rubella)^4^ have also been detected in oral swab samples. A limited number of previous studies have shown that antibodies to dengue virus (DENV) can be detected in the saliva of acute dengue patients, with a sensitivity greater than 90% compared with plasma.^7^–^9^ Only one published study has compared saliva to plasma in a community-based study of DENV incidence, which reported a sensitivity and specificity of 82% and 81% for saliva compared with plasma.^10^

Finger-prick blood samples dried onto filter paper have advantages over venous blood in that they require smaller volumes, are simple to collect by non-medically trained staff, do not require facilities for centrifugation, and can be stored and transported at ambient temperatures. The use of dried blood spots (DBS) is well established in human immunodeficiency virus (HIV) serosurveillance,^11^ viral load, and drug resistance monitoring,^12^ and for malaria serosurveillance^13^ and drug resistance studies.^14^ Previous studies have demonstrated that DENV-specific antibodies, viral RNA, and NS1 antigen can be detected in DBS samples from dengue patients,^10^,^15^–^17^ however these studies did not present systematic comparisons of the performance of DBS against matched plasma samples by assay type. Such a comparison is necessary if DBS are to be used as an alternative sample in existing diagnostic assays and algorithms for laboratory diagnosis of dengue in acute patients.

We evaluated the use of oral swab samples and DBS, compared with plasma, for the detection of antibody, viral RNA, and dengue NS1 antigen in acute dengue patients. We also evaluated oral swab samples against plasma for the detection of past DENV exposure in healthy volunteers.

## Materials And Methods

### Study population.

Oral swab samples and DBS were collected from dengue patients enrolled in existing clinical studies and from healthy volunteers enrolled in an existing serosurveillance study, from which venous blood was also collected within existing study protocols. Informed consent for the collection of oral swab and DBS samples was obtained from all participants, or their guardians in the case of pediatric patients. The study protocols were approved by the scientific and ethical committees at the Hospital for Tropical Diseases (HTD) and Tien Giang Provincial Hospital (TGH), and the Oxford University Tropical Research Ethical Committee.

Oral swab samples were collected from two populations of dengue patients. The first population were 66 children 5–18 years of age admitted to the HTD in Ho Chi Minh City (HCMC), Vietnam, between September 2009 and September 2010 with ≤ 72 hours of illness and a clinical diagnosis of dengue that was confirmed by a positive NS1 lateral flow rapid test using plasma (Bio-Rad, Hercules, CA). The second population was 50 patients admitted to TGH, southern Vietnam, between March and July 2010 with a clinical diagnosis of dengue and ≤ 72 hours of illness. In both patient populations, oral swabs and venous blood samples were collected from consecutive patients at the time of study enrollment and again before discharge. Oral swab samples and venous blood samples were also collected at a single time point from 60 healthy adult staff at the HTD in November 2009.

Venous blood samples were collected from a fourth population of 44 adults admitted to the HTD in HCMC, Vietnam, between July and October 2010 with ≤ 72 hours of illness and a diagnosis of dengue confirmed by a positive NS1 lateral flow rapid test using plasma (Bio-Rad). Samples were collected from consecutive patients at the time of study enrollment and again before discharge. Whole venous blood was spotted onto filter paper and dried as described below.

### Sample collection and processing.

A specially designed swab (Oracol Swab, Malvern Medical, Worcester, UK) was used to collect oral fluid samples, by rubbing the swab over the gum and tooth interface for 1 minute. Samples were processed on the day of collection by adding 1 mL of phosphate-buffered saline (PBS) containing 0.2% Tween-20 and 10% fetal calf serum (FCS) (PBS/0.2%Tw20/10%FCS) to the swab tube, vortexing, and then centrifuging for 2 minutes at 2,000 rpm. Using a clean pair of gloves for each sample, the eluate was manually squeezed out of the swab. Samples were stored at –20°C until assayed.

Twenty microliter spots of whole venous blood were deposited onto strips of Whatman 3 MM (Maidstone, UK) filter paper and air dried at room temperature overnight before storage in sealed bags with desiccant at 4°C. Samples were eluted into either 400 μL PBS/0.05%Tw20 (for serology), 150 μL Platelia NS1 ELISA assay diluent (Bio-Rad; for NS1 antigen detection), or 560 μL viral lysis buffer (QIAamp Viral RNA Mini kit, Qiagen, Valencia, CA; for RNA extraction and RT-PCR). The filter paper was manually agitated then shaken at 300 rpm overnight at 4°C (30 minutes at room temperature for RNA extraction) to ensure adequate sample elution, and then centrifuged to remove debris before the eluate was collected. Eluted samples were stored at –20°C until assayed, except for RT-PCR, which was performed immediately.

### Dengue diagnostics.

A capture IgM and IgG (MAC/GAC) ELISA assay using DENV/Japanese encephalitis virus antigens and mAb reagents provided by Venture Technologies (Sarawak, Malaysia) was performed with plasma samples as previously described.^18^ Plasma was used at a dilution of 1/100 and samples eluted from oral swabs and DBS were used undiluted in the same assay. The MAC/GAC ELISA Units reported represent the ratio of the sample optical density (OD), minus background OD, to a cut-off value that is five times the average OD for negative controls. Dengue viral RNA was extracted from 140 μL of plasma or the eluate from one blood spot using the QIAamp Viral RNA Mini kit (Qiagen) according to the manufacturer's instructions. An internally controlled, serotype-specific, real-time RT-PCR assay was used to detect DENV RNA as described previously.^19^ NS1 was detected using the Bio-Rad NS1 Platelia ELISA assay according to manufacturer's instructions, except that eluates from oral swabs and DBS were used undiluted.

Cases were classified as “confirmed acute dengue” according to a diagnostic algorithm (Supplementary Figure 1) based on a positive result in RT-PCR, NS1 antigen detection, and/or serology on paired plasma samples.

Evidence of past exposure to dengue virus in healthy volunteers was determined by detection of DENV-specific IgG in plasma using the PanBio Dengue IgG Indirect ELISA (Alere Inc., Waltham, MA) according to the manufacturer's instructions, except that eluates from oral swabs were used undiluted. The PanBio Units reported represent the ratio of sample ODs to a cut-off value, as specified in the manufacturer's instructions.

### Data analysis.

The sensitivity and specificity of oral swab samples or DBS, compared with plasma, was calculated for each of the diagnostic assays. The Mann-Whitney *U* test was used for comparison of medians between groups.

## Results

### Oral swab samples for diagnosis of acute dengue.

Oral swabs and matched venous blood samples were collected from 66 children admitted to the HTD in HCMC with a clinical diagnosis of dengue and a positive result in a rapid NS1 test, and from 50 children and adults admitted to the TGH with a clinical diagnosis of dengue. The difference in diagnostic criteria between the two study sites was both pragmatic, to append alternative sample collections to existing studies, and purposeful to permit an estimation of specificity by ensuring some non-dengue patients were included. Patient and sample characteristics are described in Table 1 .

Of the 116 patients enrolled, 95 had confirmed acute DENV infections. Six of 95 dengue patients had detectable DENV-specific IgM and/or IgG in enrollment plasma samples, of which two were both IgM and IgG positive (Table 2 ). DENV-specific IgM/IgG was not detectable in the corresponding oral swab sample for any of these patients. Samples were collected at the time of hospital discharge from 106 of 116 patients. Detection of DENV-specific antibodies in discharge oral swab samples was less sensitive than in plasma (Table 2), and IgG was detected with greater sensitivity than IgM in oral swab samples (91.9% versus 68.7%). The specificity of IgM and IgG detection from oral swabs was very high, and all “false positive” results were patients with laboratory-confirmed DENV infection in whom serology in the corresponding plasma sample was negative.

NS1 was detected by Platelia ELISA in oral swab samples from 55 of 85 (64.7%) patients with detectable NS1 in plasma. The specificity of NS1 detection in oral swabs was very high; the one NS1 “false positive” result was a patient with laboratory-confirmed DENV infection in whom the corresponding plasma NS1 result was equivocal. PCR was performed on enrollment oral swab samples from six participants in whom the corresponding plasma sample was PCR positive, but DENV RNA could not be amplified from any of these oral swabs so we did not continue with PCR on the remaining oral swab samples.

### DBS samples for diagnosis of acute dengue.

**Figure 1.**
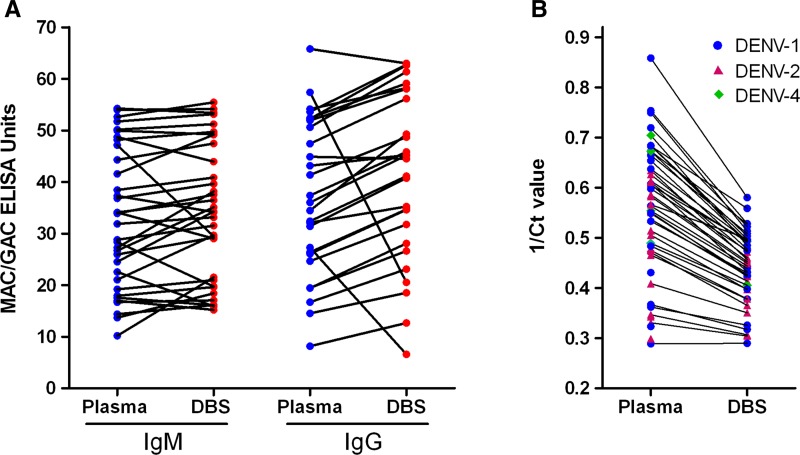
Quantitative sensitivity of dried blood spots (DBS) compared with plasma for detection of dengue virus (DENV)-specific antibody and viral RNA. (**A**) The DENV-immunoglobulin (Ig) M and -IgG capture (MAC/GAC) enzyme-linked immunosorbent assay (ELISA) Units, as a proxy for antibody quantity, are shown for paired plasma and DBS samples collected at hospital discharge from acute dengue patients. (**B**) The reciprocal of the cycle threshold (ct) value in DENV serotype-specific reverse transcription-polymerase chain reaction (RT-PCR), as a proxy for RNA quantity, is shown for paired plasma and DBS samples collected at hospital admission from acute dengue patients.

Dried blood spot samples were collected from 44 adults admitted to the HTD in HCMC with a clinical diagnosis of dengue and a positive result in a rapid NS1 test using plasma collected at enrollment. The sensitivity and specificity of IgM, IgG, NS1 (Platelia assay) and viral RNA detection from DBS relative to plasma was very high (Table 3 ). Equivocal serology results in three DBS samples with corresponding IgM/IgG-negative plasma samples were the cause of the observed less than perfect specificity in serology. When we examined the quantitative sensitivity of antibody detection from DBS compared with plasma, using OD in ELISA as a proxy for antibody quantity, we observed little or no loss of sensitivity using the DBS in the serological assays (Figure 1A). In contrast, a substantial loss of sensitivity was observed in PCR (Figure 1B), reflecting a lower level of RNA amplification from the smaller sample volume present in the DBS compared with plasma.

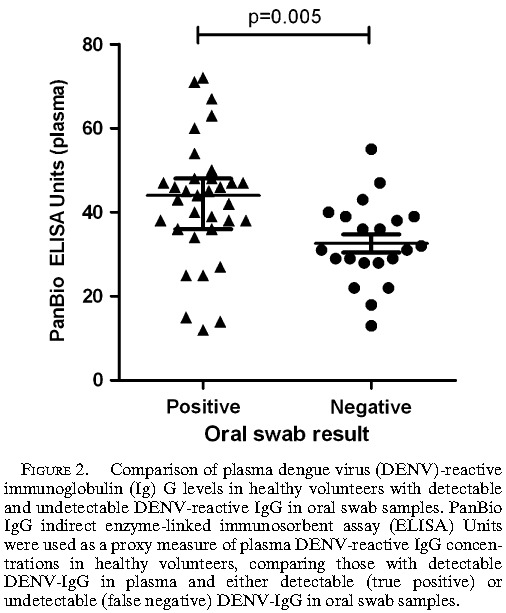



### Oral swab samples for detection of past flavivirus infection in healthy volunteers.

The performance of oral swab samples collected from 60 healthy volunteers for detection of DENV-reactive IgG compared with matched plasma samples, is shown in Table 4 . Among the 54 patients with detectable DENV-specific IgG in plasma, we compared the median plasma DENV-IgG level (using ELISA units as a proxy) between those with positive versus negative oral swab samples. The median plasma DENV-IgG level among oral swab false negatives was significantly lower than the group with detectable DENV-IgG in oral swab samples, indicating a quantitative relationship between antibody levels in plasma and oral fluid and an overall reduction in sensitivity in oral swab samples compared with plasma.

## Discussion

Oral swab samples and DBS were evaluated as alternatives to plasma for the diagnosis of acute dengue and for screening for past exposure to dengue virus. DENV-specific antibody and NS1 antigen were detected both in oral swab samples and DBS, using standard diagnostic assays. DENV RNA was amplified from DBS but not from oral swabs. There was little loss of sensitivity or specificity in using DBS compared with plasma. Oral swabs suffered from a loss of sensitivity, but retained good specificity.

Among laboratory-confirmed dengue patients, DENV-specific IgM or IgG antibodies were detectable in enrollment plasma samples in only 6% of cases, compared with 100% of discharge plasma samples. The sensitivity of IgM detection was reduced in oral swabs compared with plasma (68.7%), and varied substantially between the two study sites. The sensitivity of IgG detection was much higher (91.9%) than IgM, but again with a large difference between the study sites. Our findings fall within the range of sensitivity and specificity estimates published previously for detection of DENV-specific IgM and IgG from oral fluid samples,^7^–^10^,^20^ including both those that used a swab device and those where saliva was collected directly. We did not investigate DENV-specific IgA, but others have reported a high sensitivity and specificity for diagnosis of acute dengue using serum or DBS for IgA detection (though poor results using saliva)^10^ and a dengue IgA rapid test is commercially available (ASSURE; MP Biomedicals, Singapore). Among patients who were NS1 antigen positive in plasma samples collected at hospital admission, NS1 antigen was also detectable in the corresponding oral swab sample in almost two-thirds of patients. To our knowledge, this is the first study to show that NS1 antigen can be detected in oral fluid collected from acute dengue patients. Although there was a substantial loss of sensitivity, this provides proof of principle that early diagnosis of dengue by NS1 detection can be done from a non-invasive oral swab sample. In patients from whom a venous blood sample cannot be collected, the availability of an oral swab sample would allow a specific, although less sensitive, diagnosis of dengue to be made.

We used standard assay procedures, using undiluted eluates from oral swabs and DBS; improvements to sensitivity may be possible with further optimization of the serological and NS1 antigen assays for oral swab samples. The performance of the oral swab sample depends on adequate saturation of the swab with fluid from the tooth-gum interface, by swabbing for 1–2 minutes. Standardized training in collection technique was provided to our study staff, but the difference in sensitivity estimates between the two sites in our study may represent differences in collection method, including inadequate location or duration of swabbing.

Our findings support existing evidence that dengue virus antibodies, NS1 antigen, and viral RNA can be detected from dried whole blood eluted from filter paper.^10^,^15^–^17^ Furthermore, we show a high level of sensitivity and specificity for DBS in each diagnostic assay, compared with plasma. In IgM/IgG serology, several equivocal results among a small total number of DBS samples with corresponding negative plasma serology resulted in imperfect specificity. Ideally, we would have repeated the serology on these equivocal DBS samples to classify them as positive or negative, but because the DBS eluate is used undiluted in the assay and only one blood spot was collected per patient per assay there was insufficient specimen remaining to repeat. The high sensitivity we observed for detection of DENV RNA in DBS is comparable with other reports,^15^–^17^ although we did show a lower quantitative sensitivity in DBS than plasma, using the inverse of the assay cycle threshold as a proxy measure for RNA quantity. This is to be expected because the 20 μL of whole blood in one DBS is equivalent to ∼10 μL of plasma, compared with 140 μL of plasma used in the RNA extraction and RT-PCR. The implication of this is that DBS represent an adequate alternative to plasma for amplification of DENV RNA from acute patients with high viremia levels, but the sample volume they contain may be insufficient for detection of lower viral loads in studies of mild or asymptomatic DENV infections outside a hospital setting, unless RNA extraction can be performed on several blood spots combined from one patient. Furthermore, the patient population in which DBS were evaluated was enrolled on the basis of a positive NS1 rapid test and, because it is known that NS1-positive patients have on average higher viral loads than NS1-negative patients, it is conceivable that the sensitivity of RT-PCR that we report is higher than might be seen in a broader dengue patient population.

A possible limitation of our sample set was that the DBS were derived from venous blood samples spotted onto filter paper in the laboratory, rather than directly from finger prick. This was done to minimize the demand on patients of additional sampling, but means that our results may not reflect exactly the performance of capillary blood samples in these diagnostic assays. The one published comparison of venous and capillary blood samples in dengue diagnostics^15^ reported that DENV RNA was detectable by RT-PCR in convalescent capillary blood samples from four patients in whom the corresponding venous blood sample was negative, indicating that we might expect an equivalent or even higher sensitivity for DENV RNA detection using finger-prick blood spots than what we observed.

Seroprevalence studies of DENV exposure can provide valuable information on the spatial range and intensity of DENV transmission including, if repeated age-stratified sample sets are available, inferences on temporal changes in transmission intensity.^21^–^24^ A barrier to such studies on a large scale is the collection of blood samples from healthy individuals, particularly children. We evaluated the detection of DENV-specific IgG in oral swabs collected from healthy volunteers, as a marker of previous DENV exposure, and observed a substantial loss of sensitivity in the oral swabs, compared with plasma. Perhaps more importantly the oral swabs suffered from poor specificity, although the total number of DENV-naive volunteers was small.

In summary, we found that DBS can be used in standard serological, molecular, and antigen detection assays for diagnosis of acute dengue, with minimal loss of sensitivity or specificity compared with plasma. We found oral swabs to have inadequate sensitivity and specificity for use in population-based studies of prior DENV exposure, however their performance for detection of DENV-specific IgM, IgG, and NS1 antigen in acute patients may make them an attractive, non-invasive option where venous blood cannot be collected, or in population-based surveillance of acute or recent infection.

### Supplementary Material

Supplemental Appendix.

## Figures and Tables

**Table 1:** Characteristics of study participants

A. Evaluation of oral swab samples and dried blood spots (DBS) in acute dengue patients							
Study site	Sample evaluated	Number of samples: plasma/oral swab or DBS		Median age in years (range)	Median day of illness (range)		Laboratory-confirmed dengue
		Enrollment	Discharge		Enrollment sample	Discharge sample	
Hospital for Tropical Diseases	Oral swab	66/66	59/59	12 (6–14)	3 (2–3)	9 (6–13)	66/66
Tien Giang Hospital	Oral swab	50/49	47/48	8 (3–17)	3 (2–4)	8 (5–16)	29/50
Hospital for Tropical Diseases	Dried blood spot	44/44	43/43	22 (15–39)	3 (1–3)	8 (3–10)	44/44
B. Evaluation of oral swab samples in healthy volunteers							
Study site	Sample evaluated	Number of samples: plasma/oral swab					
Hospital for Tropical Diseases	Oral swab	60/60					

**Table 2:** Sensitivity and specificity of oral swab samples for detection of dengue virus-specific antibody and NS1 antigen in acute dengue patients

	At enrollment			At discharge		
	HTD (n = 66)	TGH (n = 49)	Total (n = 116)	HTD (n = 59)	TGH (n = 47)	Total (n = 106)
IgM						
Plasma positive (of which OS positive)	3 (0)	1 (0)	4 (0)	56 (44)	27 (13)	83 (57)
Sensitivity (95% CI)	0%	0%	0%	78.6% (67.8–89.3)	48.1% (29.3–67.0)	68.7% (58.7–78.7)
Plasma negative (of which OS negative)	62 (61)	43 (43)	105 (104)	1 (1)	20 (20)	21 (21)
Specificity (95% CI)	98.4% (95.3–100)	100%	99.0% (97.2–100)	100%	100%	100%
IgG						
Plasma positive (of which OS positive)	2 (0)	2 (0)	4 (0)	38 (37)	24 (20)	62 (57)
Sensitivity (95% CI)	0%	0%	0%	97.4% (92.3–100)	83.3% (68.4–98.2)	91.9% (85.2–98.7)
Plasma negative (of which OS negative)	63 (62)	47 (47)	110 (109)	17 (14)	22 (22)	39 (36)
Specificity (95% CI)	98.4% (95.3–100)	100%	99.1% (97.3–100)	82.4% (64.2–100)	100%	92.3% (83.9–100)
NS1						
Plasma positive (of which OS positive)†	62 (38)	23 (17)	85 (55)			
Sensitivity (95% CI)	61.3% (49.2–73.4)	73.9% (56.0–91.9)	64.7% (54.5–74.9)			
Plasma negative (of which OS negative)	0 (0)	24 (23)	24 (23)			
Specificity (95% CI)	n/a	95.8% (87.8–100)	95.8% (87.8–100)			

* OS = oral swab; 95% CI = 95% confidence interval; HTD = Hospital for Tropical Diseases; TGH = Tien Giang Provincial Hospital.

† Insufficient OS sample available for NS1 testing for four participants.

Note: except as noted in (†) where plasma positive and plasma negative do not sum to total number of samples, this is because plasma samples with equivocal results in an assay have been excluded from the calculations for that assay.

**Table 3:** Sensitivity and specificity of dried blood spots for detection of dengue virus-specific antibody, NS1 antigen and viral RNA in acute dengue patients*

	At enrollment (*N* = 44)	At discharge (*N* = 43)
IgM		
Plasma positive (of which DBS positive)	1 (1)	33 (33)
Sensitivity (95% CI)	100%	100%
Plasma negative (of which DBS negative)	42 (42)	8 (6)
Specificity (95% CI)	100%	75.0% (45.0–100)
IgG		
Plasma positive (of which DBS positive)	1 (1)	25 (24)
Sensitivity (95% CI)	100%	96.0% (88.3–100)
Plasma negative (of which DBS negative)	43 (43)	15 (14)
Specificity (95% CI)	100%	93.3% (80.7–100%)
NS1		
Plasma positive (of which DBS positive)†	43 (43)	
Sensitivity (95% CI)	100%	
Plasma negative (of which DBS negative)	0 (0)	
Specificity (95% CI)	–	
PCR		
Plasma positive (of which DBS positive)	44 (42)	
Sensitivity (95% CI)	95.5% (89.3–100)	
Plasma negative (of which DBS negative)	0 (0)	
Specificity (95% CI)	–	

* DBS = dried blood spot; 95% CI = 95% confidence interval.

† Insufficient DBS sample available for NS1 testing for one participant.

Note: except as noted in (†) where plasma positive and plasma negative do not sum to total number of samples, this is because plasma samples with equivocal results in an assay have been excluded from the calculations for that assay.

**Table 4:** Sensitivity and specificity of oral swab samples for detection of past dengue virus infection in healthy volunteers

	*n* = 60
IgG	
Plasma positive (of which OS positive)	54 (33)
Sensitivity (95% CI)	61.1% (48.1–74.1)
Plasma negative (of which OS negative)	6 (3)
Specificity (95% CI)	50% (10.0–90.0)

OS = oral swab; 95% CI = 95% confidence interval.
