# Supplementary material for: An Evaluation of Dried Blood Spots and Oral Swabs as Alternative Specimens for the Diagnosis of Dengue and Screening for Past Dengue Virus Exposure
Source: Am J Trop Med Hyg. 2012 Jul 1;87(1):165–70. doi: 10.4269/ajtmh.2012.11-0713 (PMC3391044; doi:10.4269/ajtmh.2012.11-0713)
Supplement: Supplemental Appendix. [file SD4.pdf]

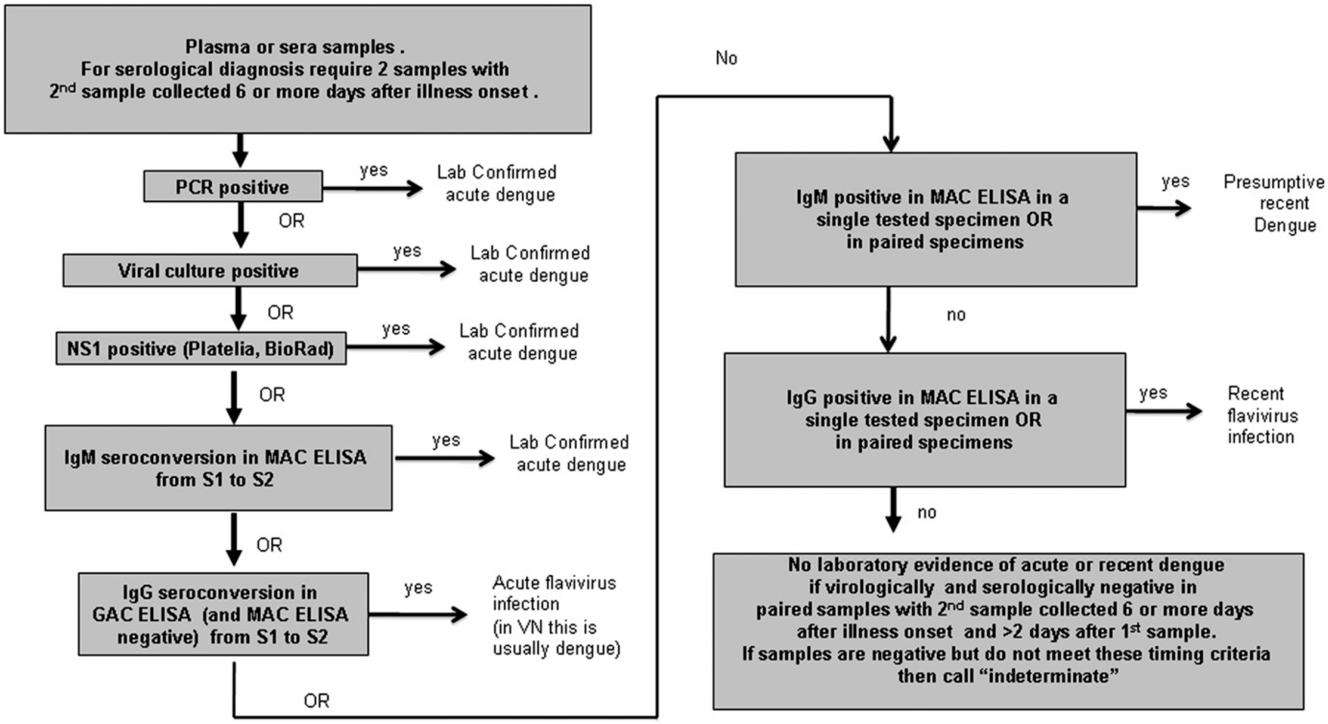

Supplemental Appendix. Dengue laboratory algorithm. In the presence of a clinical syndrome that might be dengue, the following definitions will apply. MAC = IgM capture ELISA; GAC = IgG capture ELISA.
